# Caries-preventive effect of glass ionomer and resin-based fissure sealants on permanent teeth: An update of systematic review evidence

**DOI:** 10.1186/1756-0500-4-22

**Published:** 2011-01-28

**Authors:** Steffen Mickenautsch, Veerasamy Yengopal

**Affiliations:** 1 Division of Public Oral Health, Faculty of Health Sciences, University of the Witwatersrand - 7 York Rd., Parktown/Johannesburg 2193, South Africa

## Abstract

**Background:**

This article constitutes a partial update of the original systematic review evidence by Yengopal et al. from 15 January 2008 (published in the Journal of Oral Science in 2009) with primary focus on research quality in regard to bias risk in trials. Its aim is to update the existing systematic review evidence from the English literature as to whether caries occurrence on pits and fissures of teeth sealed with either GIC or resin is the same.

**Methods:**

In addition to the 12 trials included during the original systematic review, 5 new trials were identified during the database search (up to 26 August 2010) and 2 further trials were included from a hand search and reference check. Of these, 3 trials were excluded and 16 were accepted for data extraction and quality assessment. The quality of accepted trials was assessed, using updated quality criteria, and the risk of bias was investigated in more depth than previously reported. In addition, the focus of quantitative synthesis was shifted to single datasets that were extracted from the accepted trials.

**Results:**

Twenty-six dichotomous and 4 continuous datasets were extracted. Meta-analysis and cumulative meta-analysis were used in combining clinically homogenous datasets. The overall outcome of the computed datasets suggest no difference between the caries-preventive effects of GIC- and resin-based fissure sealants.

**Conclusions:**

This overall outcome is in agreement with the conclusions of the original systematic review. Although the findings of the trials identified in this update may be considered to be less affected by attrition- and publication bias, their risk of selection- and detection-/performance bias is high. Thus, verification of the currently available results requires further high quality randomised control trials.

## Introduction

Pits and fissures of posterior teeth are considered to be highly susceptible to the adhesion of micro-organisms and consequently, to caries. Therefore, a significant amount of tooth decay occurs at these sites. Fissure sealants are used to prevent occlusal caries, 71% percent of occlusal decay being preventable after a once-off fissure sealant application [1]. Evidence regarding the efficacy and cost-effectiveness of sealants in reducing occlusal caries in molars has been highlighted [1-5]. The most commonly used sealant material is resin composite [6-8]. Its caries-preventive effect relies on the sealing of pits and fissures through micro-retention, created through tags after enamel acid etching. However, these are easily destroyed by saliva contamination, which reduces micro-retention and, consequently, the caries-preventive effect [9]. Under the generally wet conditions in the oral cavity, Glass Ionomer Cement (GIC) offers an alternative. Owing to its hydrophilic properties, GIC is not as moisture-sensitive as hydrophobic resin [10].

In a previous systematic review Yengopal et al. [11] conducted a meta-analysis in order to quantitatively appraise, for the first time, the evidence regarding the caries-preventive effect of GIC in comparison to that of resin-based fissure sealants. This systematic review with meta-analysis found no evidence that either material was superior to the other in the prevention of dental caries. Therefore, both appeared to be equally suitable for clinical application as fissure sealant materials. These results were based on a systematic search of literature up to 15 January 2008 [11]. It has been suggested that once the search date of a systematic review is older than even 1 year, users should check for more recent trials on the same topic to see whether new evidence has altered the findings of a given systematic review [12]. In addition, the original quality assessment criteria [11] may be questioned on grounds of being ineffective in judging the true internal validity of trials on basis of risk of bias [13,14]. Therefore, the aim of this update is to provide a more in-depth assessment of bias-risk in trials. As the inclusion of non-English language trials in the original systematic review did not decisively influence the overall review results [11] the focus of the in-depth assessment and discussion of bias-risk is limited to English language trials, only.

Thus, the purpose of this article is to update the existing evidence from trials published in English language regarding the review question as to whether caries occurrence on pits and fissures of teeth sealed with either GIC or resin is the same.

## Materials and methods

In order to update the existing evidence, the systematic literature search of the English literature was extended beyond the original search date and a further hand search and reference check were done. The quality of accepted trials was assessed, using updated quality criteria (Table 1) [13-16] and the risk of bias was investigated in more depth than previously reported. In addition, the focus of quantitative synthesis was shifted to single datasets (DS) that were extracted from the accepted trials.

**Table 1:** Quality assessment criteria of trials

Selection bias		
**Score**	**Criteria**	**Impact on bias risk**
Randomisation and concealment		
A	(i) Randomisation: Details of any adequate type of allocation method that generates random sequences with the patient as unit of randomisation are reported.^1^	Doubts may still exist whether the trial results are influenced by selection bias but no indication can be found from the trial report to support such doubt.
	(ii) Concealment: Trial provides evidence^2 ^that concealment was indeed effective and that the random sequence could not have been observed or predicted throughout the duration of the trial.	
B	(i) Randomisation: Details of any adequate type of allocation method that generates random sequences with the patient as unit of randomisation are reported.^1^	Despite the implementation of method considered to be able to prevent unmasking of the concealed allocation sequence through direct observation and prediction, there are reasons to expect that the concealed allocation sequence may have been unmasked during the cause of the trial.
	(ii) Concealment: Trial reports on any adequate method to prevent direct observation^3 ^and prediction^4 ^of the allocation sequence and sequence generation rules.	
C	(i) Randomisation: Details of any adequate type of allocation method that generates random sequences with the patient as unit of randomisation are reported.^1^	Despite the implementation of method considered to be able to prevent unmasking of the concealed allocation sequence through direct observation, there are reasons to expect that operators could have predicted the concealed allocation sequence.
	(ii) Concealment: Trial reports on any adequate method to prevent direct operator observation of allocation sequence and sequence generation rules^3^. However, the allocation sequence and sequence generation may have been sufficiently predicted.	
D	(i) Randomisation: Details of any adequate type of allocation method that generates random sequences with the patient as unit of randomisation are reported.^1^	Despite the theoretical chance for each patient to be allocated to either treatment group, operator knowledge of the allocation sequence may have lead to patient allocation that favoured the outcome of one type of treatment above the other.
	(ii) Concealment: The trial report does not include information on how the allocation of random sequence was concealed. The allocation could have been directly observed and/or predicted.	
0	Trial does not comply with criteria A - D.	No guaranty of equal chance for patients to be allocated to either treatment group, thus allocation may have favoured the outcome of one type of treatment above the other.
Baseline data for randomised trials		
A	Baseline data collected before randomisation and reported for both treatment groups. Data shows no significant differences between both groups.	Evidence is given that randomisation has lead to equal groups suggesting little risk of selection bias.
B	Baseline data collected before randomisation and reported for both treatment groups. Data shows significant differences between both groups but has been statistically adjusted appropriately.	Differences have been adjusted, thus the influence of possible selection bias appears to be reduced.
C	Baseline data collected before randomisation and reported for both treatment groups. Data shows significant differences between both groups without being statistically adjusted.	Reported differences may be due to ineffective randomisation, thus indicate risk of selection bias.
0	Trial does not comply with criteria A - C.	No evidence is given whether randomisation has indeed lead to equal groups with differences beyond chance, thus differences may exists indicating selection bias.
Detection/Performance bias		
Blinding/Masking		
Score	Criteria	Impact on bias risk
A	(i) Trial reports on any type of method that is known to prevent patient AND operator AND evaluator to discern whether patients are allocated to the test- or the control group (Blinding/Masking).	Evidence is given that the trial results may not have been influenced by detection/performance bias that may have favored the outcome of one type of treatment above the other.
	(ii) Trial reports a process with which the effect of Blinding/Masking was evaluated, as well as the results of such evaluation.	
B	(i) Trial reports on any type of method that is known to prevent patient AND operator AND evaluator to discern whether patients are allocated to the test- or the control group (Blinding/Masking).	Doubts may still exist whether the trial results are influenced by detection/performance bias but no indication can be found from the trial report to support such doubt. However, no evaluation of the Blinding/Masking effect has been included in the trial, thus no evidence for lack of bias is given.
	(ii) Trial report does not give reason for doubt that the patient allocation to either the test- or the control group has been unmasked throughout the duration of the trial.	
C	(i) Trial reports on any type of method that is known to prevent patient AND operator AND evaluator to discern whether patients are allocated to the test- or the control group (Blinding/Masking).	Despite the implementation of method considered to be able to prevent unmasking, there are reasons to expect that operators/patients could have discovered the allocation.
	(ii) Trial report gives reason for doubt that the patient allocation to either the test- or the control group has been unmasked throughout the duration of the trial.	
0	No process reported or implemented able to blind/mask patients AND operators whether patients where allocated to either the test- or the control group (It is insufficient to report that blinding/masking was done without reporting the details of the process).	Knowledge about the patient allocation may have caused patients/operator to act in a way that may have favoured the outcome of one type of treatment above the other,
Attrition bias		
Loss - to follow up		
Score	Criteria	Impact on bias risk
A	Available case analysis, loss-to-follow up reported per treatment group. Subsequent sensitivity analysis does not indicate a possible risk of bias.	The trial allows extracting evidence that attrition may not have favoured the outcome of one type of treatment above the other.
B	Available case analysis, loss-to-follow up reported per treatment group. Subsequent sensitivity analysis indicates a possible risk of bias.	The trial allows assessing the risk that attrition may have favoured the outcome of one type of treatment above the other.
0	Trial does not report number of included participants per treatment group at baseline or gives any indication that would allow ascertaining the loss-to-follow up rate per treatment group.	The trial carries an unknown risk that attrition may have favoured the outcome of one type of treatment above the other.
Run-in phase		
A	No run-in phase reported or discernable during which patients were given the active treatment or the placebo/control.	The trial may not carry the risk of bias due to exclusion of patients who would not respond well to e.g. the active treatment.
0	Run-in phase reported or discernable during which patients were given the active treatment or the placebo/control.	During a run-in phase only patients were selected for randomisation that have responded/not responded to the active treatment of the placebo/control. This may favour the outcome of one type of treatment above the other as patients who did not respond well to either are excluded.
Trial endpoints		
0	The trial reports on secondary or surrogate outcomes as endpoints.	Even if the surrogate results would highly correlate with primary (i.e. clinical) outcomes, they cannot serve as valid replacements and need to be regarded for hypothesis development, only.
A	The trial reports on primary outcomes as endpoints.	Primary outcomes may provide evidence for hypothesis testing.

^1 ^Excluded are types of allocation methods that are considered as inadequate: cluster randomisation, fixed block randomisation with block size 2, minimization, alternation, randomisation of teeth, use of date of birth or patient record number, "quasi"-randomisation, split-mouth

^2 ^E.g. by reporting results of the Berger-Exner Test or any other statistical tests that show that covariates of compared groups were similar at baseline

^3 ^E.g. by opening of opaque envelope, obtaining allocation from tables, computer generated or from other sources

^4 ^E.g. central randomisation, sequence allocation by other than operator; excluding varied block randomisation

### Literature search, review and quality assessment of trials

The search strategy used in the previous review for English language articles [11] was replicated for this review update using the search terms: "(GIC sealant* OR Glass ionomer cement sealant) AND (caries OR tooth decay)". Only the start and cut-off dates were changed. The English databases, Biomed Central, Cochrane Oral Health Reviews, Cochrane Library, Directory Of Open Access Journals, Expanded Academic ASAP PLUS, Meta Register Of Controlled Trials, PubMed and Science-Direct, were searched for relevant papers published between 15 January 2008 (the search cut-off date of the original systematic review) and 26 August 2010. Criteria for trial inclusion were:

- 2-or multiple arm clinical prospective study designs;

- Comparison of GIC versus Resin fissure sealants;

- Publication in English.

Included trials were excluded after further review if:

- No outcome measure related to caries was reported;

- No computable data, dichotomous or continuous, per treatment group was reported;

- Resin-modified GIC (RMGIC) was used instead of conventional, chemically cured GIC.

Included trials that passed the exclusion criteria test were accepted for further quality assessment and data extraction. Reviewing, data extraction and quality assessment of the accepted trials was undertaken independently by two reviewers (SM and VY). Differences were resolved through discussion and consensus.

Unlike in the original published systematic review [11], quality assessment of accepted trials was undertaken on the basis of availability of evidence indicating successful prevention of selection- and detection/performance bias from the start to end of each trial. The new criteria (Table 1) differed from those previously used in the first review [11]. It has been argued that the inclusion of bias-preventing measures (e.g. randomisation, blinding/masking) into the trial methodology only demonstrates an attempt to reduce bias risk but does not carry proof in itself that such attempt was indeed successful and that it is far more important to judge trial quality according to evidence that indicates to what extent such attempt has succeeded [13]. Against this background the quality criteria were adjusted accordingly. For example; if a trial merely reported that randomisation was conducted; reported only the name of the randomisation method used or included a detailed description of the randomisation process without providing any evidence that randomisation was indeed effective throughout the trial; then this was regarded as inadequate.

Potential attrition- and publication bias was not investigated in the original meta-analysis [11]. In this update, sensitivity analysis was done, using the RevMan Version 4.2 statistical software of The Nordic Cochrane Centre, The Cochrane Collaboration (Copenhagen; 2003), in order to investigate potential attrition bias risk in trials. To investigate publication bias a funnel plot was generated, using the datasets from the included clinical trials. The standard error (SE) of the mean differences was plotted on the Y-axis, and the log of the Relative Risk (RR) on the X-axis, using MIX Version 1.7 meta-analysis software [17]. In addition, Egger's linear regression method [18] was used to calculate an intercept with a 95% Confidence Interval (CI), with statistical significance set at α = 0.05.

We anticipated that many of the identified trials would be of split-mouth design. The split-mouth study design is commonly used in dentistry to test interventions and has the advantage of having an individual serve as both the experiment and control. In this study design, one or more pairs of teeth (e.g. primary molars) form the unit of randomisation. Strictly, these pairs are not independent and should be analysed as "paired data" on a patient basis. However, similar to other systematic reviews where split-mouth trials are included [2] we analysed the pairs of tooth surfaces independently for differences whether they are carious or not. The fact that this would result in slightly narrower confidence intervals was considered in the discussion of the overall results.

### Data extraction and analysis

All data concerning primary and secondary outcomes of accepted trials were extracted either as single dichotomous datasets (containing the number of observed effects (n) and the total number of evaluations (N) for both the control and the test groups) or as single continuous datasets (containing the mean value, standard deviation and total number of evaluations for both the control and the test groups). For dichotomous datasets the Relative Risk (RR, 95% CI), and for continuous datasets the Mean Difference (MD, 95% CI), was computed, using the Cochrane RevMan, Version 4.2 software package. Statistical significance was set at α = 0.05.

Meta-analysis, using RevMan Version 4.2 statistical software by The Nordic Cochrane Centre, The Cochrane Collaboration (Copenhagen; 2003), was considered for datasets only if they complied with criteria for clinical homogeneity. Datasets were considered to be clinically homogeneous if they covered the same type of dentition; type of teeth; study length; type of evaluation method; type of GIC (high- or low viscosity) and stated whether the resin-based fissure sealant material contained fluoride. The percentage of total variations across datasets (I^2^) was used in assessing statistical heterogeneity [19]. Statistical significance for assessing statistical heterogeneity was set at α = 0.10. A fixed effects model was used for meta-analysis under condition of statistical homogeneity of datasets (p > 0.10) and a random-effects model was used for the others. Pooled datasets were assigned a Mantel-Haenszel weight directly proportionate to the sample size.

Cumulative meta-analysis, using MIX Version 1.7 meta-analysis software [17], was performed on datasets of consecutive follow-up periods, in order to investigate whether a trend of the available evidence might be observed in line with increasing time after sealant application. Care was taken to select only clinically homogenous datasets. A random-effects model was used under condition of statistical heterogeneity of datasets (p < 0.10).

## Results

### Literature search

**Figure 1:**
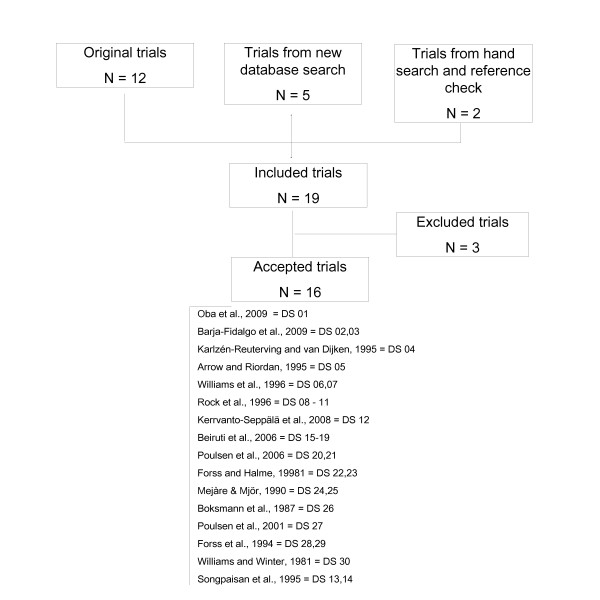
**Flow diagram of trial selection**. N = Number of trials; DS = Dataset number.

In addition to the 12 trials included during the original systematic review [20-31], five trials [32-36] were identified during the new database search. A further two trials [37,38] from the hand search and reference check were included (Figure 1). Of these 19 trials, 3 were excluded [34-36], owing to lack of reported caries outcome [35] and reporting on RMGIC as a test material [34,36]. Sixteen trials passed the exclusion criteria and were accepted for data extraction and quality assessment [20-33,37,38].

### Dataset extraction and analysis

Twenty-six dichotomous (DS 01-12,15-28,30) and 4 continuous datasets (DS 13,14,23,29) were extracted from the 16 accepted trials. Characteristics of these trials and their datasets are shown in Table 2. The articles by Forss et al. [37] and Forss and Halme [28] reported each of different datasets from the same trial. Three of the accepted 16 trials followed a parallel group design [25,26,33], while all other trials were split-mouth studies.

**Table 2:** Details of accepted trials

Article	DS	Study design	GIC treatment group					Resin treatment group					Outcome measure	Evaluation		Dentition/Teeth/Restoration	Study period
					
			Type of material	BSL	N	n	LTF	Type of material	BSL	N	n	LTF		Criteria	Method		
		Dichotomous datasets															
Oba et al., 2009 [32]	01	SM	Ketac Molar	91	56	6	35	Fissurit F	116	81	8	35	Caries	Caries present	Visual	First permanent molars	3 years
Barja-Fidalgo et al., 2009 [33]	02	PG	Fuji IX	46	46	1	0	Delton	46	46	2	0	Caries	Cavity that had clearly penetrated the dentin or if a radiolucency in dentin could be seen on the bitewing X- ray	Visual and X-Ray	First permanent molars	6 months
	03			46	21	2	25		46	28	7	18					5 years
Karlzén-Reuterving and van Dijken, 1995 [20]	04	SM	Fuji III	74	72	1	2	Delton	74	72	3	2	Caries	Caries present	Visual	First permanent molars	3 years
Arrow and Riordan, 1995 [21]	05	SM	Ketac Fil	465	412	6	53	Delton	465	412	31	53	Caries	When a cavity was present	Visual	First permanent molars	Mean 3.64 (SD 0.11) years
Williams et al., 1996 [22]	06	SM	Fuji III	430	295	19	135	Delton	430	295	4	135	Caries	Caries present	Visual	First permanent molars	2 years
	07			430	222	22	208		430	222	16	208					4 years
Rock et al., 1996 [23]	08	SM	Baseline	172	160	3	12	Fluoro- Shield	172	162	0	10	Caries	Caries present	Visual	First permanent molars	6 months
	09			172	157	6	15		172	158	1	14					1 year
	10			172	130	14	42		172	132	2	40					2 years
	11			172	124	18	48		172	129	3	43					3 years
Kerrvanto- Seppälä et al., 2008 [24]	12	SM	Fuji III	1025	657	27	368	Delton	1025	657	7	368	Caries	If dentine caries was detected	Visual	2^nd ^permanent molars	3 years
Beiruti et al., 2006	15	PG	Fuji IX	180	180	0	0	Visio Seal	180	180	1	0	Caries	Caries present	Visual	First permanent molars	1 year
[26]	16			180	154	0	26		180	161	6	19					2 years
	17			180	154	3	26		180	138	13	42					3 years
	18			180	143	7	37		180	123	21	57					4 years
	19			180	80	8	100		180	76	27	104					5 years
Poulsen et al., 2006 [27]	20	SM	Fuji III	364	364	34	0	Delton	364	364	10	0	Radiograp-hically carious	Lesions into dentine	X-Ray	First permanent molars	2.3 - 3.2 years
	21			364	364	23	0		364	364	10	0	Clinically carious	Danish municipal dental service criteria	Visual		
Forss and Halme, 1998^1 ^[28]	22	SM	Fuji III	166	97	23	69	Delton	166	97	16	69	Caries	Caries lesion present/Arrested caries present	Visual	Permanent molars/premolars	7 years
Mejàre and Mjör, 1990 [29]	24	SM	Fuji III	44	36	0	8	Delton	117	75	6	42	Caries	Caries present	Visual	Permanent molars/premolars	5 years
	25			44	36	0	8	Concise	47	18	2	29					
Boksmann et al., 1987 [30]	26	SM	Fuji III	125	116	0	9	Concise	122	115	0	7	Caries	Caries present	Visual	Permanent molars	6 months
Poulsen et al., 2001 [31]	27	SM	Fuji III	170	116	44	54	Delton	170	116	13	54	Caries	White, yellow, brown discoloration of the fissure or cavity	Visual	First permanent molars	3 years
Forss et al., 1994^1 ^[37]	28	SM	Fuji III	166	151	7	15	Delton	166	151	7	15	Caries	Caries lesion present/Arrested caries present	Visual	Permanent molars/premolars	2 years
Williams and Winter, 1981 [38]	30	SM	ASPA	No info	486^2^	64^2^	No info	Concise	No info	486^2^	93^2^	No info	Caries	Caries present	Visual	First permanent molars	3.84 years
Article	DS	Continuous datasets															
		Patient character-istics/potential confounders*	Type of material	N	x	SD	LTF	Type of material	N	x	SD	LTF	Outcome measure	Criteria	Method	Dentition/Teeth/Restoration	Study period
Songpaisan et al., 1995 [25]	13	PG	Fuji III	128	0.48	1.03	14%	Delton	133	0.05	0.57	14%	DFS increment	DFS	Visual	Permanent molars	2 years
	14			128	1.82	2.60			133	0.98	1.72		DMFS increment	DMFS			
Forss and Halme, 1998^1 ^[28]	23	SM	Fuji III	97	0.13	0.40	69	Delton	97	0.13	0.37	69	Caries increment on approximal tooth surfaces adjacent to materials	Caries lesion present/Arrested caries present	Visual	Permanent molars/premolars	7 years
Forss et al., 1994^1 ^[37]	29	SM	Fuji III	151	0.09	0.34	15	Delton	151	0.13	0.41	15					2 years
Article	Place of trial	Age of patients	Patient characteristics/inclusion/exclusion criteria	Fluoride exposure from other sources													
Oba et al., 2009 [32]	Study conducted in a boarding school in the city of Kırıkkale/Turkey	7-11 years	Children received instructions on good oral health care and were individually shown how to clean their teeth prior to the start of the treatment; inclusion criteria were: (1) sound pits and fissures in fully erupted first molars; and (2) pits and fissures diagnosed with an early enamel lesion; exclusion criteria were: (1) partly erupted first molar; (2) an obvious cavity in the occlusal surface; and (3) the presence of a restoration or a sealant (or part of it) in the pit and fissure system;													Resin-based fissure sealant material containing fluoride	
Barja-Fidalgo et al., 2009 [33]	Study carried out in the Department of Paediatric Dentistry, Rio de Janeiro State University, Rio de Janeiro/Brazil;	mean age 6.8 years (±0.98 SD)	With at least 1 permanent first molar erupted and 2 or more primary molars decayed, filled, or extracted due to caries; most from low socio-economic background with high caries-risk; the participants were also given oral hygiene instructions and dietary counselling; those who had dental care needs were referred to the paediatric dental clinic for appropriate restorative and surgical treatment; participants reported brushing their teeth daily, 11% reported using dental floss regularly, and 67% had a dental check-up once a year first molars that presented a sound occlusal surface or occlusal caries at the D1 level (non-cavitated enamel lesion) entered the study; low patient compliance and high saliva contamination during treatment reported.													-	
Karlzén-Reuterving and van Dijken, 1995 [20]	Children from Umea/Sweden	mean age 7 years, 1 month	Teeth without clinical evidence of caries were sealed													-	
Arrow and Riordan, 1995 [21]	Children from Perth/Australia	mean age 7 (SD 0.72) years	With sound, unsealed, homologous 1st permanent molars													-	
Williams et al., 1996 [22]	Children from Suffolk/UK	6-8 years	Recently erupted visually caries free 1st permanent molars; resealed teeth were excluded													Fluoride concentration of drinking water 0.1 - 0.5 mgF/l	
Rock et al., 1996 [23]	Children from Tamworth, Staffordshire/UK	7-8 years	Caries free fully erupted 1st permanent molars; children had evidence of caries in primary teeth													The resin-based sealant FluroShield contains fluoride; fluoride concentration of drinking water 0.13 ppm	
Kerrvanto- Seppälä et al., 2008 [24]	Children from Varkaus/Finland	12-16 years	2nd permanent molars considered to be at risk of caries were sealed; teeth with lost resin sealant were resealed and not excluded from the study.													-	
Beiruti et al., 2006 [26]	Children from Damascus/Syria	mean age 7.8 years	No cavities in primary teeth; inclusion criteria: sound pits and fissures in fully erupted 1st permanent molars, pits and fissures with early enamel lesion and/or small dentinal lesion; exclusion criteria: partly erupted tooth, obvious occlusal cavity, presence of restoration or sealant in pits and fissures.													-	
Poulsen et al., 2006 [27]	Children from Vaerlose/Denmark	8-13 years	Mean DMFS was between 0.5 and 0.7 for 12-year-old children and between 1.4 and 1.8 for 13-year-old children; sound surfaces, and surfaces with initial or arrested caries (white or brown fissures) were sealed, if the dentist's clinical assessment indicated a caries risk; only children with both clinical and radiographic data were included in the present study													Fluoride content of the drinking water 0.25 ppm; all children commonly use fluoridated toothpaste	
Forss and Halme, 2006 [28]	Children from Raisio/Finland	5-14 years	Contra-lateral pair of newly erupted non-sealed permanent molars or premolars													-	
Mejàre and Mjör, 1990 [29]	-	mean age 9.2 (5.7 - 15.0) years	-													-	
Boksmann et al., 1987 [30]	-	6-18 years	Patients had not received topical fluoride treatment for at least 3 months prior.													Fluoride content of the drinking water 1 ppm or more	
Poulsen et al., 2001 [31]	Children from Damascus/Syria	7 years	Only children with at least one pair of caries free permanent 1st molars or with incipient lesions; average DMFT 0.6 -0.7.													-	
Forss et al., 1994 [37]	Children from Raisio/Finland	5-14 years	Contra-lateral pair of newly erupted non-sealed permanent molars or premolars													-	
Williams and Winter, 1981 [38]	-	6-8 and 11-13 years	-													-	
Songpaisan et al., 1995 [25]	Children from Bangkok/Thailand	12-13 years	From very low to medium socio-economic background; children with at least 3 sound permanent molars (erupted sufficiently)													Included in a fluoride mouth rinse programme (0.2% NaF) every 2 weeks; fluoride concentration of drinking water 0.1 - 0.2 ppm	

DS = Dataset number; BSL = Number of teeth at baseline; N = Number of teeth evaluated; n = Number of teeth with caries, LTF = Loss-to-follow-up; x = Mean; SD = Standard deviation; PG = Parallel group design; SM = Split-mouth design.

^1 ^Different datasets reported from same trial

^2 ^Number of pits reported, instead of the number of sealed teeth

* Potential confounders = Reported fluoride exposure; high-sugary diet; poor oral hygiene; high past caries experience.

Sixteen of the 30 datasets showed no difference between the two materials after periods lasting from 6 months to 7 years (Table 2 and 3). Twelve dichotomous (DS 05, 10,11,17-21,27,30) and two continuous datasets (DS 13,14) showed statistically significant (p < 0.05) different results. Of these, seven dichotomous datasets (DS 06,10-12,20,21,27) extracted from five trials [22-24,27,31] were in favour of resin-based fissure sealants after 2 to 3 years. Five dichotomous datasets (DS 05,17-19,30) extracted from three trials [21,26,38] were in favour of GIC-based fissure sealants after 3 to 5 years. Two continuous datasets (DS 13,14) from one trial [25] reporting on secondary outcomes, such as the DFS and DMFS increment, were also in favour of resin-based fissure sealants after 2 years.

Three of the datasets with statistically significant results (DS 17-19) were derived from one trial including high-viscosity GIC [26].

### Meta-analysis

**Figure 2:**
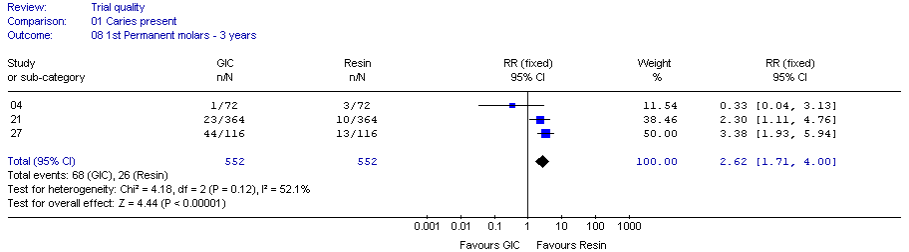
**Forrest plot of meta-analysis results concerning caries on sealed first permanent molars after 3 years**. Study or sub-category = Dataset number; GIC = Glass-ionomer cement; RR = Relative Risk; CI = Confidence Interval; n = Number of teeth with caries; N = Total number of evaluated teeth.

**Figure 3:**
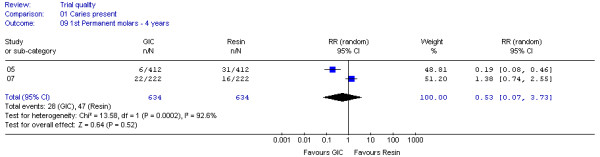
**Forrest plot of meta-analysis results concerning caries on sealed first permanent molars after 4 years**. Study or sub-category = Dataset number; GIC = Glass-ionomer cement; RR = Relative Risk; CI = Confidence Interval; n = Number of teeth with caries; N = Total number of evaluated teeth.

Of the 30 datasets, two groups of datasets, DS 04,21,27 after 3 years and DS 05,07 after 4 years, were considered to have each met the criteria for clinical homogeneity and were combined in two meta-analyses. The results were generated in the form of two forest plots (Figure 2 and 3). These datasets included first permanent molars sealed either with low-viscosity GIC or with resin material lacking fluoride and were evaluated by visual, clinical examination.

Figure 2 shows a pooled relative risk of 2.62 (95% CI: 1.71 - 4.00; p < 0.00001), suggesting a 2-3 times higher chance of caries for teeth sealed with low-viscosity GIC than for those filled with resin, after 3 years. Additional analysis established a low risk of statistical heterogeneity (I^2 ^= 56.7%, p = 0.13). For that reason a fixed-effects model was used for this meta-analysis.

Figure 3 shows a pooled relative risk of 0.53 (95% CI: 0.07 - 3.73; p = 0.52), suggesting no difference between the chance of caries development in teeth sealed with low-viscosity GIC or resin after 4 years. Additional analysis established a high risk of statistical heterogeneity (I^2 ^= 92.8%, p = 0.0002). For that reason a random-effects model was used for this meta-analysis. The significant statistical heterogeneity may be related to the inconsistency in the size of the treatment effects as the trials from which both datasets were extracted were similar in type of materials, sample size, outcome measure, evaluation criteria and method, as well as type of dentition, teeth, age of patients and study period (Table 2). Further investigation, by using the available trial information, did not result in any other possible explanation.

### Cumulative meta-analysis

**Figure 4:**
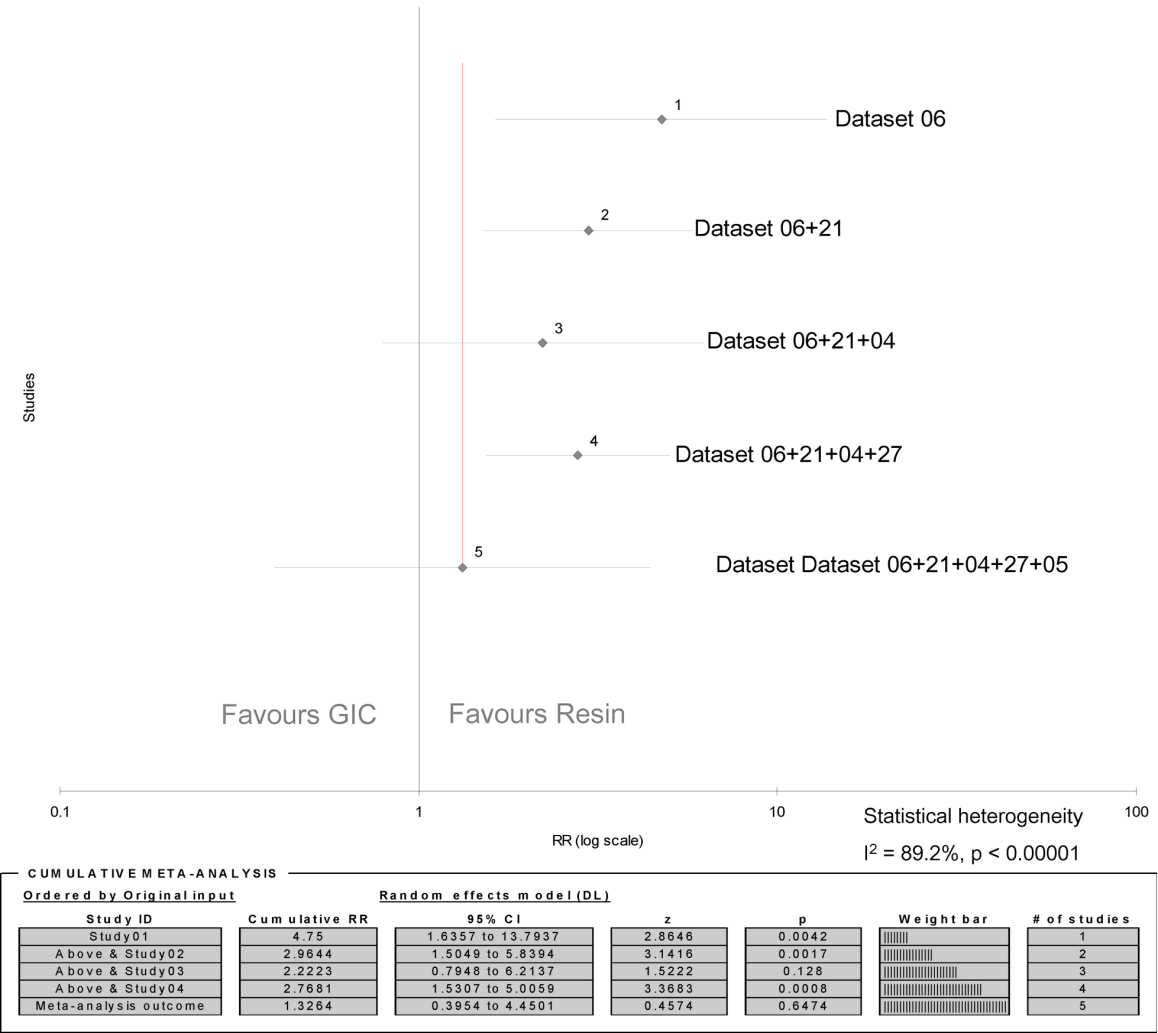
**Forrest plot of cumulative meta-analysis results concerning caries on sealed first permanent molars**. RR = Relative Risk

In order to investigate whether a possible trend may be assumed in the comparison of GIC and resin, the chronological results from 5 datasets, concerning sealed teeth after 2 years (DS 06), 2-3 years (DS 21), 3 years (DS 04,27) and 3.64 years (DS 05) [20-22,27,31], were included in a cumulative meta-analysis and its results were generated in the form of a forest plot (Figure 4). As with both meta-analyses (Figure 2 and 3), all datasets included first permanent molars sealed either with low-viscosity GIC or resin material without fluoride and were evaluated by visual, clinical examination. They were thus considered clinically homogenous in all aspects except their follow-up periods. However, a statistical heterogeneity was established (I^2 ^= 89.2%; p < 0.00001) that may be attributed to the differences in the length of follow-up periods, so a random-effects model was used. The cumulative Relative Risk indicates no statistical significant difference between the two materials after 5 years (RR 1.33; 95% CI: 0.39 - 4.45; p = 0.65). A shift of the cumulative relative risks towards the value of 1.00, as well as a shift of the cumulative 95% confidence intervals below a relative risk of 1.00 from the period after 2 to 3.64 years can be observed (Figure 4).

### Quality assessment of trials and risk of bias

#### Selection-, Detection-/Performance bias risk

The results of the quality assessment regarding selection- and detection/performance bias are shown in Table 4. None of the accepted trials reported sufficient details of any randomisation process that had indeed given each patient the same chance to be allocated to either the GIC or the resin group and to ensure that direct observation and prediction of the allocation sequences was successfully prevented. Only three trials [25,26,33] had reported baseline data collected before randomisation and reported for both treatment groups, statistically compared this data between groups and found the difference statistically not significant (p > 0.05). No accepted trial reported on successful blinding/masking of patients, operators and trial evaluators.

#### Attrition bias risk

Sensitivity analysis was used in computing all datasets, under the assumption that either:

(i) All pits and fissures of sealed teeth lost to follow-up developed caries;

(ii) None of the sealed teeth lost to follow-up developed caries.

The results of either situation did not change the conclusions for the majority of the datasets. However, a possible risk of attrition bias was identified for the results of four datasets (DS 06,12,24,25) extracted from three trials [22,24,29]. Under the assumption that all pits and fissures of sealed teeth lost to follow-up developed caries, the results of two datasets (DS 06,12) would not be statistically significantly in favour of resin: DS 06 - RR 1.11 (95% CI: 0.92 - 1.33; p = 0.28); DS 12 - RR 1.05 (95% CI: 0.94 - 1.18; p = 0.36) and the results of two datasets (DS 24,25) would be statistically significantly in favour of GIC: DS 24 - RR 0.44 (95% CI: 0.23 - 0.86; p = 0.02); DS 25 - RR 0.28 (95% CI: 0.14 - 0.53; p = 0.0001).

In line with the potential influence of attrition bias on datasets, the meta-analysis results (Figure 2 and 3) were not affected.

Under the assumption that all pits and fissures of sealed teeth lost to follow-up developed caries, the results of the cumulative meta-analysis (Figure 4) would only change towards a further shift of the cumulative relative risks towards the value of 1.00, and in a narrowing of the cumulative 95% confidence interval after 3.64 years (RR 1.14 - 95% CI: 0.081 - 1.60; p = 0.46).

In addition to the risk of bias due to loss-to-follow up, no trial indicated that a run-in phase was implemented before randomisation (Table 4).

#### Publication bias risk

**Figure 5:**
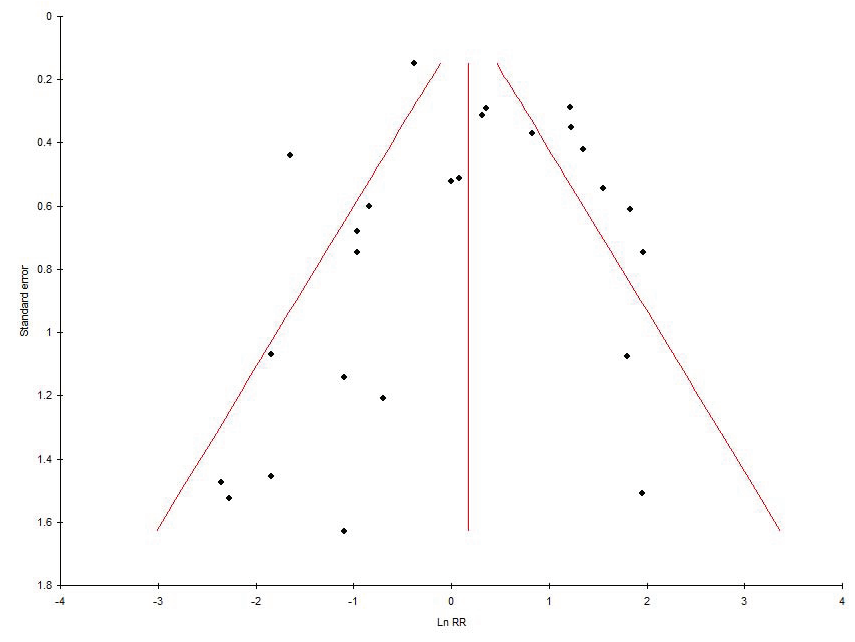
**Funnel plot of dataset results (test for publication bias)**. RR = Relative Risk

Publication bias was investigated, using one funnel plot (Figure 5). The funnel plot concerning data for caries progression showed an even distribution that did not suggest publication bias. Egger's linear regression method for the same datasets showed an intercept of 0.05 (95% CI: -1.74, 1.85; p = 0.95). The regression result was not statistically significant.

Unlike the original systematic review, non-English language articles were not included in this partial update and this needs to be addressed in a future update, including not only those in Portuguese and Spanish but in other languages too.

## Discussion

The aim of this article was to update the existing evidence from trials published in English language regarding the review question as to whether caries occurrence on pits and fissures of teeth sealed with either GIC or resin is the same. The article constitutes a partial update of the original systematic review evidence with primary focus on research quality in regard to bias risk in trials.

During the new systematic literature search seven more trials [32-38] could be included for review. The reason for this was that five new trials had been published since the cut-off date of the original search; a more thorough hand search and reference check of the literature had been done, and broader inclusion criteria were used. Of the seven new included trials, three were excluded [34-36] as they did not comply with the stated exclusion criteria.

In comparison to the original published systematic review [11], this update presents an improvement in the output of its systematic search of English trials. However, other aspects in the methodology of this review update might still have contributed to limitations in its results: (i) not all relevant publications were listed in the selected databases; (ii) The chosen search terms may not have been broad enough; (iii) not all relevant publications could be found through a hand search and reference check. In addition and as this update is limited to English language trials, only, a future update of the non-English evidence of the original systematic review is required. Although, the inclusion of non-English language trials in the original systematic review did not decisively influence the overall review results [11] an update of the non-English evidence of the original systematic review would confirm (i) whether the new findings from non-English trials substantially differ from the English language trials and (ii) whether results from any new non-English language trials would influence the overall review results.

### Selection-, Detection-/Performance bias risk

Of the accepted 16 trials, only 3 trials followed a parallel group design [25,26,33] and all other were split-mouth studies. Mejáre et al. have cautioned against the split-mouth design as "randomised", as the common practice of including subjects with at least one pair of caries-free molars results in exclusion of caries-active subjects [39]. An obvious selection bias is thus created, as not all subjects will have the same chance to participate. Mejáre et al. have rightfully suggested that the split-mouth trial design should therefore be regarded as "quasi-randomised". Thus, reviews where inclusion criteria include only randomised-control trials should, in theory, exclude trials that use the split-mouth study design. Following the example of other systematic reviews [2], the data from split-mouth trials was analysed as independent data. This will have caused narrower confidence intervals and thus may have favoured the reported outcomes of one type of treatment above the other. However, the so caused differences in confidence intervals may be considered to only be slight [2] and its correction would not have affected the general impact of selection bias on the relative risk (RR, 95% CI) per dataset (Table 3) due to lack of adequate randomisation [39-41]. In addition, wider confidence intervals would have provided only stronger indication of no difference between both types of treatment and would not have changed the overall conclusion from the current analysis results.

**Table 3:** Results of individual datasets

Article	DS	Dichotomous datasets		
		
		RR	95% CI	p-value
Oba et al., 2009 [32]	01	1.08	0.40 - 2.96	0.87
Barja-Fidalgo et al., 2009 [33]	02	0.50	0.05 - 5.32	0.57
	03	0.38	0.09 - 1.65	0.20
Karlzén-Reuterving and van Dijken, 1995 [20]	04	0.33	0.04 - 3.13	0.34
Arrow and Riordan, 1995 [21]	05	0.19	0.08 - 0.46	0.0002*
Williams et al., 1996 [22]	06	4.75	1.64 - 13.79	0.004**
	07	1.38	0.74 - 2.55	0.31
Rock et al., 1996 [23]	08	7.09	0.37 - 136.11	0.19
	09	6.04	0.74 - 49.58	0.09
	10	7.11	1.65 - 30.66	0.009**
	11	6.24	1.89 - 20.66	0.003**
Kerrvanto- Seppälä et al., 2008 [24]	12	3.86	1.69 - 8.79	0.001**
Beiruti et al., 2006 [26]	15	0.33	0.01 - 8.13	0.50
	16	0.08	0.00 - 1.42	0.08
	17	0.21	0.06 - 0.71	0.01*
	18	0.29	0.13 - 0.65	0.003*
	19	0.28	0.14 - 0.58	0.0006*
Poulsen et al., 2006 [27]	20	3.40	1.71 - 6.78	0.0005**
	21	2.30	1.11 - 4.76	0.02**
Forss and Halme, 1998^1 ^[28]	22	1.44	0.81 - 2.55	0.21
Mejàre and Mjör, 1990 [29]	24	0.16	0.01 - 2.73	0.20
	25	0.10	0.01 - 2.03	0.14
Boksmann et al., 1987 [30]	26	Not estimable		
Poulsen et al., 2001 [31]	27	3.38	1.93 - 5.94	<0.0001**
Forss et al., 1994^1 ^[37]	28	Not estimable		
Williams and Winter, 1981 [38]	30	0.69	0.51 - 0.92	0.01*
Article	DS	Continuous datasets		
		MD	95% CI	p-value
Songpaisan et al., 1995 [25]	13	0.43	0.23, 0.63	<0.0001**
	14	0.84	0.30, 1.38	0.002**
Forss and Halme, 1998^1 ^[28]	23	0.00	-0.11, 0.11	1.00
Forss et al., 1994^1 ^[37]	29	-0.04	-0.12, 0.04	0.36

DS = Dataset number; RR = Relative risk; MD = Mean difference; CI = Confidence interval; Not estimable = data from both treatment groups are essentially the same: p = 1.00.

* Statistically significant difference, in favour of GIC

** Statistically significant difference, in favour of Resin

^1 ^Different datasets reported from same trial

All of the accepted trials appear to be limited by risk of selection- and detection-/performance bias. Bias or systematic error may affect studies, causing either an over- or under-estimation of the treatment effect of an investigated clinical procedure. Overestimation has been observed to be the most common [40]. Kjaergard et al. reported a treatment effect overestimation of 48% caused by lack of random sequence allocation [41] and Egger et al. reported a treatment effect overestimation of 54% and 53% due to lack of allocation concealment and lack of evaluator blinding, respectively [42].

It has been emphasized that selection bias can only be successfully prevented if the allocation sequence remains truly random and free from potential interference throughout the trial [13,14]. Thus, it is important that trials should include an effective process for concealing the random allocation sequence and that by the end of each trial this process has indeed prevented direct observation and prediction of the random sequence allocation [13,14]. Quality assessment in terms of the internal validity of trials should therefore be a measure of the result of random sequence allocation and allocation concealment, and not only of its reported attempt. All trials accepted in this systematic review failed to report not only on evidence of successful sequence allocation and allocation concealment results, but also on necessary details about how sequence allocation and allocation concealment were attempted (Table 4). None of the trials, therefore, provide any guarantee that each patient had an equal chance of being allocated to either treatment group and thus, their allocation may have favoured the outcome of one type of treatment above the other. One measure for testing whether random sequence allocation has not been successful is testing whether covariates differ between treatment groups at baseline [13]. Only three articles had included such a test and reported on its outcome [25,26,33]. The statistically non-significant results (p > 0.05) suggest a successful random allocation (Table 4). However, doubt remains regarding potential bias risk, as other non-balanced covariates may exist, that were not tested for and/or not reported.

**Table 4:** Results of quality assessment of accepted trials

Article	DS	Selection bias		Detection/Performance bias	Attrition bias		Trial outcome
			
		Randomization	Baseline data	Blinding/Masking	Loss-to-follow up	Run-in phase	
Oba et al., 2009 [32]	01	0	0	0	A	A	A
Barja-Fidalgo et al., 2009 [33]	02	0	A	0	A	A	A
	03	0	A	0	A	A	A
Karlzén-Reuterving and van Dijken, 1995 [20]	04	0	0	0	A	A	A
Arrow and Riordan, 1995 [21]	05	0	0	0	A	A	A
Williams et al., 1996 [22]	06	0	0	0	B	A	A
	07	0	0	0	A	A	A
Rock et al., 1996 [23]	08	0	0	0	A	A	A
	09	0	0	0	A	A	A
	10	0	0	0	B	A	A
	11	0	0	0	A	A	A
Kerrvanto-Seppälä et al., 2008 [24]	12	0	0	0	B	A	A
Beiruti et al., 2006 [26]	15	0	A	0	A	A	A
	16	0	A	0	A	A	A
	17	0	A	0	B	A	A
	18	0	A	0	B	A	A
	19	0	A	0	A	A	A
Poulsen et al., 2006 [27]	20	0	0	0	A	A	A
	21	0	0	0	A	A	A
Forss and Halme, 1998^1 ^[28]	22	0	0	0	A	A	A
Mejàre and Mjör, 1990 [29]	24	0	0	0	B	A	A
	25	0	0	0	B	A	A
Boksmann et al., 1987 [30]	26	0	0	0	A	A	A
Poulsen et al., 2001 [31]	27	0	0	0	A	A	A
Forss et al., 1994^1 ^[37]	28	0	0	0	A	A	A
Williams and Winter, 1981 [38]	30	0	0	0	0	A	A
Songpaisan et al., 1995 [25]	13	0	A	0	n.e.	A	0
	14	0	A	0	n.e.	A	0
Forss and Halme, 1998^1 ^[28]	23	0	0	0	n.e.	A	0
Forss et al., 1994^1 ^[37]	29	0	0	0	n.e.	A	0

DS = Dataset number, n.e. = Not evaluated.

^1 ^Different datasets reported from same trial

From the onset, in all trials successful blinding or masking appeared not to have been possible, owing to the obvious differences in clinical appearance between GIC and resin sealants. For that reason the allocation to either treatment group was visible to patients, operators and evaluators. However, the difficulties of successful blinding still carry the danger of detection-/performance bias, which may thus have affected the trials' results. Potential knowledge of superiority claims prior to the trial may have led patients to change their oral hygiene habits, operators to place restorations more carefully or evaluators to apply evaluation criteria more subjectively. This in turn may have favoured the outcome of one type of treatment over the other.

### Attrition bias risk

Sensitivity analysis may be used in establishing whether missing data could have affected trial outcomes by assuming that the numbers of restoration lost to evaluation were either failures or successes [43]. Comparison of the analysis results with reported trial outcomes indicates whether different conclusions should be drawn. Sensitivity analysis was conducted for all datasets. The analysis results differed from reported outcomes of four datasets (DS 06,12,24,25) extracted from three trials [22,24,29]. According to the analysis results, more datasets (DS 24,25) would have been in favour of GIC and fewer in favour of resin if all sealed teeth that were lost to follow-up were assumed to have developed caries in their pits and fissures. How high the caries rate in the teeth lost to evaluation really was remains unknown. Owing to uncertainty regarding the real caries prevalence within those lost to follow-up, the results of the sensitivity analysis cannot serve as evidence that GIC would perform better than resin. However, the validity of the four datasets (DS 06,12,24,25) can be questioned on grounds of attrition bias and thus, their results need to be regarded with caution.

None of these datasets were included into the meta-analyses (Figure 2 and 3) and thus do not affect their results. However, one dataset (DS 06) was also included in the cumulative meta-analysis (Figure 4). A re-computation of the data including all loss-to-follow-up (under assumption of caries) did not change the initially observed trend. Rather, the trend was reinforced, as the change from a cumulative RR after 3.65 years of 1.33 to 1.14 would suggest.

A run-in phase is considered to be a stage during a trial where all patients receive, for example, the test treatment and only those patients that respond well to the treatment are later used for random allocation in either the control or the test group [14]. Such practice would effectively exclude patients from the randomisation process and potentially favour one of the groups. No such run-in phase was indicated in any of the accepted trials (Table 4).

### Publication bias risk

Publication bias was investigated by generating a funnel plot (Figure 5). Publication bias is present when the results of published research differ from those of all the studies that have been done [44]. Funnel plots are scatter graphs showing the size of studies on the Y-axis (large studies on top; small studies at the bottom) and the effect size, observed in these studies, on the X-axis. The effect sizes of larger studies have the tendency to cluster near the mean. Small studies have effect sizes that are dispersed across a wider range. Results of both types of studies, plotted on a scatter graph, give the shape of an inverted, in absence of publication bias, symmetrical funnel [45]. Publication bias affects a funnel plot in the form of a concentration of studies to one side only (asymmetry). Such asymmetry is created when particular smaller studies are published only when they show a larger than average effect. However, if the number of studies (n) is less than ten, any asymmetry may be due to chance and not to publication bias [46]. For that reason the decision was made to plot results of the 26 extracted dichotomous datasets as units of investigation. These are not all independent from the published trials and this forms a departure from the common application of funnel plots in investigating for publication bias. Despite this departure, the use of datasets (instead of published trials) will also indicate potential publication bias when only datasets that show a larger than average effect are published and other datasets are not. In our update, the funnel plot concerning dichotomous data on caries progression showed a symmetrical spread of dataset results (Figure 5). As the visual judgement of funnel plots is subjective, we calculated intercepts (95% CI), using Eggers regression [18]. The calculated non-significant intercept confirmed the observations from the funnel plot. Both suggest that any potential impact of publication bias may be low in regard to this topic.

### Data extraction and analysis results

The extended scope of this update did not change the overall results of the original published systematic review [11]. However, it has to be noted that these results are limited by risk of selection- and detection-/performance bias. As the true extent of such bias impact remains unknown within the reviewed trials, the results need to be regarded with caution.

Five of the accepted studies reported on fluoride exposure of subjects from drinking water [22,23,25,27,30], a fluoride-rinsing programme [25] and toothpaste [27]. Two trials compared GIC fissure sealants with resin materials that reportedly contained fluoride [23,32]. In addition, most of the trials followed a split-mouth design, providing the possibility of fluoride release from the GIC fissure sealants into the oral cavity. This may have provided a caries-preventive effect for the resin-sealed teeth. It can be assumed that this may have increased caries resistance and thus confounded a potential caries-preventive effect of GIC, as suggested by Hara et al. [47]. Notwithstanding any possible confounder impact, the overall results suggest that resin-based materials may be more caries-preventive after 2-3 years of sealant placement (Figure 2). Such benefit does not translate beyond the duration of 3 years as Figure 3, as well as the majority of datasets without statistically significant outcomes (Table 3), suggests. Other statistically significant outcomes in favour of resin from datasets (DS 06,12), not included in the meta-analysis, may be invalid because of attrition bias and datasets of non-significant outcomes (DS 24,25) could very well have shown statistically significant outcomes in favour of GIC if all studied teeth could have been evaluated at the end of the trials. All these observations suggest that weakness exists in any claims of resin superiority over GIC and further, rather suggest that the caries-preventive effects of both types of material are equal.

These considerations should be regarded as hypothetical, owing to the high risk of selection/detection/performance bias in all accepted trials. Plausibility for this conclusion is provided by the results of the included cumulative meta-analysis (Figure 4). Cumulative meta-analysis is used to provide insights into how much the efficacy of treatments, often reported as mean results with 95% confidence intervals, change over time as evidence accumulates [48]. It is the result of conducting a new meta-analysis each time a new set of evidence emerges [49]. A cumulative meta-analysis not only allows evaluation of any additional contributions made by individual studies to the cumulatively combined results of preceding studies [50] but also allows observation of a trend in the direction of evidence over time. The pooling of datasets of different study periods may be questioned on the grounds that their heterogeneity (due to differences in the length of observation periods) could render the result of a meta-analysis meaningless. On the other hand, it can be assumed that datasets covering longer study periods insert a larger weight into the cumulative results and thus generate a potential trend concerning treatment effects observed after increasing periods of time. The length of an observation period is related to the trial outcome as with its length also the length of, e.g. exposure to cariogenic factors increases. Thus the weight of trial outcomes after a longer observation period would be larger. Such period effect may be investigated through cumulative meta-analysis because it allows for the pooling of outcomes from (otherwise clinically homogeneous) trials after continuously increasing time periods. Should the period effect be stronger for one of the compared treatment types than the other, a trend in the direction of the cumulated point estimates and confidence intervals can be shown by use of a forest plot. Such cumulative results may not provide **proof **in support of a particular hypothesis due to prevailing statistical heterogeneity. However, the results may assist in providing **plausibility **for hypothesis development. The forest plot in Figure 4 shows a shift of the cumulative results towards the value of RR 1.00, thus indicating that 'no difference' between low-viscosity GIC and resin may be a more plausible hypothesis with increasing time after sealant application than the hypothesis that 'resin is superior to GIC'. Such hypothesis requires thorough testing through further high quality randomised control trials.

The results of two datasets (DS 17,19) from trials in favour of high-viscosity GIC after 3 and 5 years [26] could not be repeated in later trials [32,33]. However, the results of the latter two trials may not be comparable with the former, as Beiruti et al. did not report on annual results that were independent from those of each previous year in this trial [26].

### Recommendations for further research

Systematic reviews have been reported to provide the highest form of clinical evidence [51]. However, the internal validity of such evidence can only be as good as the internal validity of the trials reviewed. Although the trials accepted in this update may be considered to be less affected by attrition- and publication bias, their risk of selection- and detection-/performance bias is high. Overestimation due to bias may have affected particularly those datasets that indicated a statistically significant difference between both material groups (Table 3) by increasing (or decreasing) the value of point estimates and by artificially narrowing their confidence intervals. However, the precise effect of bias on all results remains unknown. Thus the results need to be regarded with caution and require verification. For that reason, further high quality randomised control trials (RCT) are needed. Such RCTs should adopt a parallel group design and include randomisation and allocation concealment methods that can effectively prevent direct observation and prediction of the allocation sequence. For this purpose, the maximum randomisation method has been suggested [13]. Covariates of both treatment groups should be tested as to whether they differ at baseline (after randomisation). Recently, inclusion of the Berger-Exner test has been suggested, to enable authors of trials to investigate whether selection bias has been introduced into their studies [13,14]. Where bias risk has been found, it may be adjusted statistically [13]. Both outcomes should be included in the final trial report. In order to ensure that the lack of blinding may not have led to favouring one treatment over the other, trials should use and report on procedures and tests employed that may limit or at least monitor potential bias risk. In addition to these recommendations, future trials should base their reporting on the CONSORT statement [52].

## Conclusion

In conclusion, the current evidence to this topic may be considered to be less affected by attrition- and publication bias. However, its risk of selection, detection and performance bias is high. Consequently, further high quality randomised control trials are needed in order to answer the question whether caries occurrence on pits and fissures of teeth sealed with either GIC or resin is the same, more conclusively.

## Competing interests

The authors declare that they have no competing interests.

## Authors' contributions

Both authors contributed equally to the systematic literature search, review, data extraction and to the writing of the manuscript. SM conducted the data analysis.
